# Mapping of the complement C9 binding domain on *Trichinella spiralis* paramyosin

**DOI:** 10.1186/1756-3305-7-80

**Published:** 2014-02-24

**Authors:** Xi Zhao, Yuwan Hao, Jing Yang, Yuan Gu, Xinping Zhu

**Affiliations:** 1 Department of Parasitology, School of Basic Medical Sciences, Capital Medical University, Beijing, China

**Keywords:** *Trichinella spiralis*, Paramyosin, Immune evasion, Complement C9, Binding domain

## Abstract

**Background:**

Trichinellosis is an important foodborne zoonosis that is distributed worldwide. *Trichinella spiralis* may evade host complement-mediated attack by expressing complement inhibitory proteins, such as paramyosin (Pmy). Previous studies have shown that *Trichinella spiralis* paramyosin (*Ts*-Pmy) is able to bind to the human complement component C9 to inhibit the complement activation and protect the parasite from complement-mediated attack. Further determination of the complement-binding domain on *Ts*-pmy will enable us to better understand the *Ts*-Pmy’s biofunction in the immune evasion and provide feasible approach to develop epitope-based subunit vaccine against trichinellosis.

**Methods:**

The complement C9 binding region on *Ts*-Pmy was determined by expression of overlapped fragments of *Ts*-Pmy and their binding activities to C9. The exact binding site was further narrowed-down to a 14-amino acid peptide at C-terminus using synthesized peptides with different size of amino acid sequence. The C9 complement-binding of the 14-amino acid peptide and its interference in the C9 polymerization and the complement-mediated lysis of rabbit erythrocytes was investigated.

**Results:**

The protein interaction between human C9 and native *Ts*-Pmy was further confirmed by immunoprecipitation with *T. spiralis* lysates. The fragmental expression and C9 binding assays identified that the binding region of *Ts*-Pmy to C9 is located within 831–885 of *Ts*-Pmy C-terminus. The exact binding site on *Ts*-Pmy to C9 was narrowed down to 14 amino acid residues (^866^Val-^879^Met) by using different sizes of synthesized peptides. In the presence of the synthesized 14-amino acid peptide, human C9 polymerization and the hemolytic activity of the human complement was inhibited.

**Conclusions:**

Our results revealed the precise molecular basis for *T. spiralis* to produce *Ts*-Pmy as an immunomodulator to evade the attack of the host complement system as a survival mechanism.

## Background

Trichinellosis is a globally widespread foodborne zoonosis that occurs by ingesting raw or undercooked meat of infected animals that contain parasitic larvae [1]. Muscle larvae (ML) are released from muscle tissue by digestive enzymes in the stomach and migrate to the small intestine, where the larvae develop into adult worms (AD). Adult females produce newborn larvae (NBL), which then penetrate into the mesenteric lymphatic vessels or the bloodstream and spread throughout the body. Migrating NBL leave the capillaries and finally invade into muscle tissue, where the NBL develop to ML and encapsulate in individual skeletal muscle cells [2].

*Trichinella spiralis* is the most common species that infects human and mammalian hosts, such as pigs [3]. Human trichinellosis is characterized by high fever, facial edema and myositis, which could be serious, particularly in elderly patients [4]. Being regarded as an emerging or re-emerging disease in some parts of the world due to changes in diet and cooking practices [3,5], trichinellosis is not only a public health hazard but also an economic problem for livestock production and food safety [6,7]. Consequently, there is an urgent requirement for developing therapeutic and preventive vaccines to control the infection [8].

The complement system represents a cornerstone of the innate defense against infection and provides a vital first line of defense against invading pathogens [9]. Among the complement-evasion strategies to escape the host immune attack, the capture of host complement components on the parasite surface and then inactivating their functions is an evasion mechanism that is frequently adopted by many parasites during the establishment of parasitism [10]. Similar to other parasitic helminthes, *T. spiralis* utilizes molecules or structures on the outermost cuticle/epicuticle to bind complement components, such as C1q, C3, C5, C8 and C9 [11-13], to evade the complement attachment by inhibiting the formation of the membrane attack complex (MAC) [13].

Paramyosin is a dimeric fibrillar protein that forms the thick myofilaments of invertebrate muscle. In our previous study, a full-length cDNA encoding *T. spiralis* paramyosin (*Ts*-Pmy) was cloned by immunoscreening an adult worm cDNA library using *T. spiralis*-infected rabbit sera [14]. BALB/c mice vaccinated with recombinant *Ts*-Pmy (r*Ts*-Pmy) developed a Th1/2 mixed immune response and were partially protected against a *T. spiralis* larval challenge [14,15]. Subsequently, the expression of *Ts*-Pmy was observed on the outer membrane of newborn larvae and adult worms using immunogold electron microscopy and immunofluorescence staining [16]. A functional analysis identified that r*Ts*-Pmy was able to bind to the human complement components C8 and C9, which consequently inhibits the formation of MAC and thereby protects the parasite from being attacked by activated complement [16]. However, the complement binding site on *Ts*-Pmy has not been determined. In the present study, we expressed different fragments of *Ts*-Pmy, characterized the interaction between different fragments of *Ts*-Pmy and the complement component C9 and finally pinpointed the complement binding site on *Ts*-Pmy within the region from ^866^Val to ^879^Met at the C-terminus.

## Methods

### Ethics statement

Experimental animals were purchased from the Laboratory Animal Services Center of Capital Medical University (Beijing, China). Experimental procedures were reviewed and approved by the Capital Medical University Animal Care and Use Committee and were consistent with the NIH Guide for the Care and Use of Laboratory Animals.

### Parasites and antigen preparation

*T. spiralis* of ISS 533 strain was maintained in female ICR mice and *T. spiralis* ML were recovered from the muscles of infected mice by a standard pepsin/hydrochloric acid digestion method [17]. Adult worms were collected from the intestines of an infected Wistar rat. Crude somatic extracts of adult worms were prepared by homogenizing the worms in 1× PBS, pH 7.4 and centrifuging at 16,000 × g. The protein concentration of the extracts was determined by BCA assay kit (Merck, Germany).

### Immunoprecipitation

To further verify the protein interaction between the human complement C9 and the native *Ts*-Pmy, immunoprecipitation was performed with *T. spiralis* adult extracts as described previously [18]. Protein G MicroBeads (Miltenyi Biotec, Germany) were pre-incubated with 3 μg of human C9 (Merck, Germany) and 2 μg of anti-C9 mAb (IgG1, Abnova, Taiwan) for 30 min on ice. In total, 40 μg of *T. spiralis* adult extracts was then added and incubated overnight at 4°C with rotation. Beads were washed four times with washing buffers (1% NP40, 50 mM Tris buffer, pH 8.0) before being added with pre-heated 1× SDS gel loading buffer to elute proteins. Samples were separated by SDS-PAGE and probed with anti-*Ts*-Pmy mAb (7E2) [19]. IRDye 800CW-labeled goat anti-mouse IgG (LI-COR, Germany) was used as the secondary antibody.

### Fragmental expressions of *Ts*-Pmy in *E. coli*

To identify and locate the binding site of *Ts*-Pmy that binds to the human complement C9, the DNA encoding the N-terminus (*Ts*-Pmy1-315), C-terminus (*Ts*-Pmy571-885) and middle region (*Ts*-Pmy286-600) of *T. spiralis* paramyosin with 30 amino acids overlapped were cloned into the bacterial expression vector pET28a, with 6-histidine expressed at the N-terminus as tags (Merck, Germany). To further locate the binding site at the C-terminus of *Ts*-Pmy, the DNA encoding the fragments of *Ts*-Pmy571-695, *Ts*-Pmy666-790, *Ts*-Pmy761-885, *Ts*-Pmy761-815, *Ts*-Pmy796-850 and *Ts*-Pmy831-885 were subcloned into pET28a to express the fragments of *Ts*-Pmy C-terminus. The recombinant plasmids with sequencing confirmed right reading frames were transformed into competent *E. coli* BL21 pLysS cells, and the recombinant *Ts*-Pmy fragments were induced with 1 mM IPTG at 37°C for 6 h. The expressed fragments were purified from the induced bacterial lysates using Ni-charged His-Bind columns (Merck, Germany). The purified fragments were examined by Western blot with a monoclonal anti-HisTag antibody (0.2 μg/ml; Merck, Germany).

### Peptide synthesis

To finally determine the amino acid sequence that binds to C9, the peptides with different amino acid sequences within the C9 binding region (^831^Leu to ^885^Tyr) were synthesized by solid-phase peptide synthesis (Aviva Bio, China). The obtained peptides were purified up to 95% by preparative RP-HPLC and verified by mass spectrometry.

### Binding assay of r*Ts*-Pmy fragments and synthesized peptides to human C9

To determine whether the expressed recombinant *Ts*-Pmy fragments bind to human C9, the fragments and non-relevant control BSA (Sigma, USA) (2 μg each) were subjected to SDS-PAGE under reducing conditions and then transferred to a nitrocellulose membrane. After blocking with 5% milk in PBS, the membrane was incubated with human C9 (Merck, Germany) (1 μg/ml) at 37°C for 2 h and then probed with anti-C9 mAb (0.2 μg/ml; Abnova, Taiwan) at room temperature for 1 h. IRDye 800CW-labeled goat anti-mouse IgG (50 ng/ml; LI-COR, Germany) was used as the secondary antibody.

To determine whether synthesized peptides bind to human C9, the peptides and non-relevant control BSA (Sigma-Aldrich, USA) (5 μg in 2.5 μl each) were spotted onto a nitrocellulose membrane using a narrow-mouth pipette tip. After blocking with 1% BSA in PBS, the membrane was incubated with human C9 (1 μg/ml) at 37°C for 2 h and then with anti-C9 mAb (0.2 μg/ml) at room temperature for 1 h.

All membranes mentioned above were detected and imaged using an Odyssey infrared imaging system (LI-COR, Germany).

### C9 polymerization assays

To determine the effect of the synthesized peptide on C9 polymerization, human C9 (3 μg) was pre-incubated with the synthesized peptide at 37°C for 40 min and then incubated with 50 mM ZnCl_2_ in 20 mM Tris buffer (pH 7.2) at 37°C for 2 h [20]. Zn^2+^-induced polymerized C9 (polyC9) is resistant to dissociation by boiling in 1% SDS and, thus, could be detected by SDS–PAGE [21]. The inhibition of C9 polymerization was shown by SDS-PAGE on a 3 to 20% acrylamide gradient gel under reducing conditions and stained with Coomassie blue. The full-length r*Ts*-Pmy was added as a positive control. Recombinant *Ts*87 (r*Ts*87), a specific *T. spiralis* secreted protein [22], was used as a non-relevant control.

### Hemolytic assay of the alternative complement pathway

The complement-mediated lysis of rabbit erythrocytes (E_R_) was performed via the alternative pathway of complement activation [12]. To identify whether the synthesized peptide acts as an inhibitor or a neutralizer of the complement-mediated lysis activated by the alternative pathway, 100 μl of fresh normal human serum (NHS) was pre-incubated with various amounts of synthesized peptide or r*Ts*-Pmy in Mg-EGTA solution (5 mM MgCl_2_, 10 mM EGTA) for 30 min before being added into freshly prepared E_R_ (1 × 10^8^) in 100 μl HBSS (Hank’s Balanced Salt Solution, without calcium and magnesium, pH 7.4, Gibco, USA) at 37°C for 30 min. Heat-inactivated NHS was used as a control. The hemolytic assay was stopped by adding 1 ml of cold HBSS that contained 10 mM EDTA. After centrifugation at 3,000 × g and 4°C for 10 min, the amount of hemoglobin released into the supernatant was measured at 412 nm. The percentage of lysis (relative to cells completely lysed by water) was then calculated. r*Ts*87 was used as a non-relevant control.

### Statistical analysis

The data were expressed as the mean ± standard error (S.E.) and were evaluated using the software Prism 6 (GraphPad Inc., USA) with a one-way ANOVA; P <0.05 was regarded as statistically significant.

## Results

### Binding of native *Ts*-Pmy to human C9

**Figure 1:**
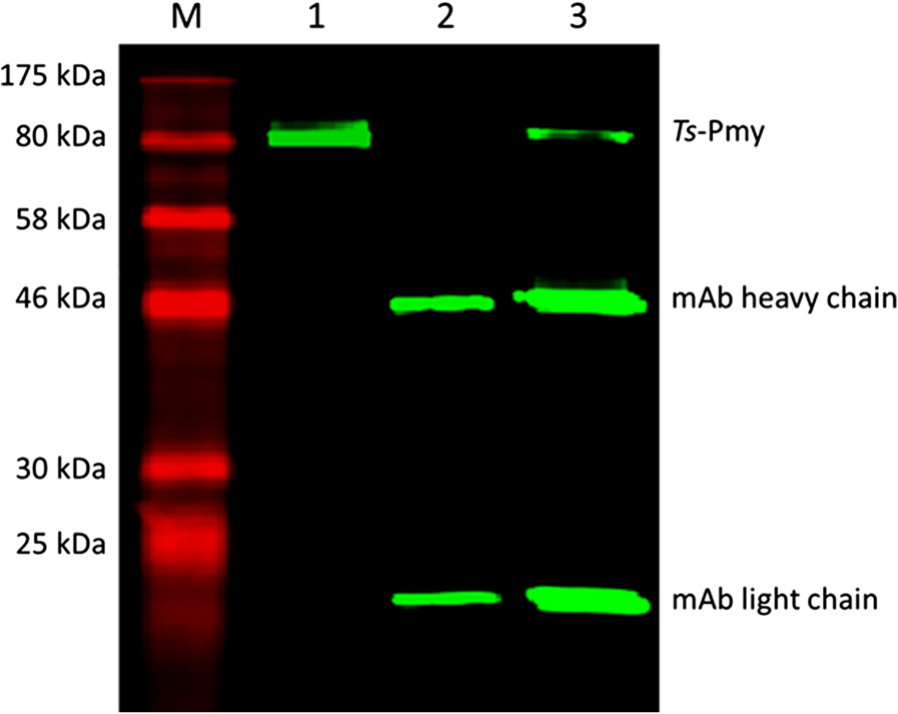
**Binding of native *Ts*-Pmy to human C9 was identified using immunoprecipitation.** The extracts of *T. spiralis* adult worms were incubated with human C9 and anti-C9 mAb and precipitated with protein G beads. The binding complex was separated by 12% SDS-PAGE and probed with anti-*Ts*-Pmy mAb 7E2. M, standard protein marker; Lane 1, *T. spiralis* adult worm extracts only; Lane 2, worm extracts incubated with anti-C9 mAb only as a control; Lane 3, worm extracts incubated with human C9 and anti-C9 mAb.

The protein interaction between human C9 and native *Ts*-Pmy was further confirmed by immunoprecipitation and Western blot analysis. *Ts*-Pmy in the adult worm extracts was specifically recognized by anti-*Ts*-Pmy mAb 7E2 (~102 kDa) (Figure 1, Lane 1). The native *Ts*-Pmy in the adult extracts was bound to human C9, and the binding complex was pulled down by anti-C9 mAb (Figure 1, Lane 3). Anti-C9 mAb alone did not bind to the native *Ts*-Pmy in the extracts (Figure 1, Lane 2). As shown in lanes 2 and 3, the heavy chain and light chain of anti-C9 mAb were pulled down by Protein G and recognized by the anti-mouse IgG secondary antibody.

### Fragmental expression mapping of the *Ts*-Pmy binding site to human C9

**Figure 2:**
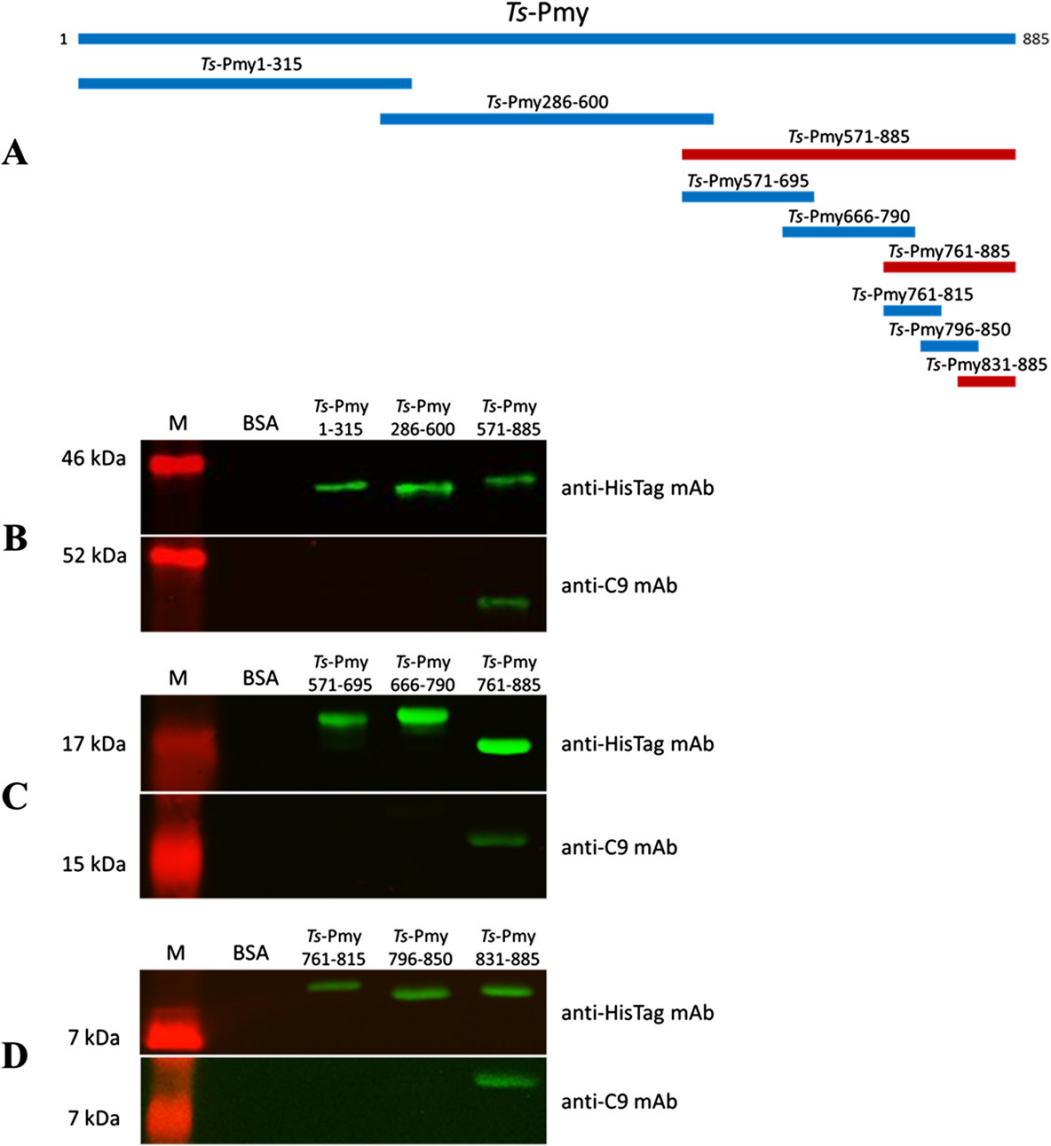
**Diagram of r*Ts*-Pmy fragments and mapping of the *Ts*-Pmy binding site to human C9.** To identify the binding site of *Ts*-Pmy to human C9, each fragment (with starting and ending positions) was cloned and expressed as a recombinant protein **(A)**. Each recombinant protein (2 μg) starting from large fragments **(B)** down to smaller fragments **(C and D)** were subjected to SDS-PAGE and then probed with anti-HisTag mAb (upper) or bound to human C9 and probed with anti-C9 mAb (lower). IRDye 800CW-labeled goat anti-mouse IgG (50 ng/ml) was used as the secondary antibody. The same amount of BSA (2 μg) was loaded as a non-relevant control. M: standard protein marker.

To identify the binding site of *Ts*-Pmy to human C9, different fragments covering the whole molecule with 30 amino acids overlapped were expressed as recombinant fragmental proteins (Figure 2A). The recombinant fragments were transferred to a nitrocellulose membrane, probed with human C9 and detected using anti-C9 mAb. The Western blot analysis demonstrated that the binding site in *Ts*-Pmy to human C9 was located at the C-terminus of *Ts*-Pmy (*Ts*-Pmy571-885) (Figure 2B). Further fragment expressions were performed within the C-terminus and the binding site was narrowed down to *Ts*-Pmy761-885 (Figure 2C). Further pinpoint of the C9 binding region was determined by smaller fragment expression, which is located within *Ts*-Pmy831-885 (Figure 2D). As a non-relevant control, BSA did not bind to human C9. All recombinant protein fragments could be recognized by anti-HisTag antibody.

### Determination of the C9 binding site in *Ts*-Pmy by synthesized peptide mapping

**Figure 3:**
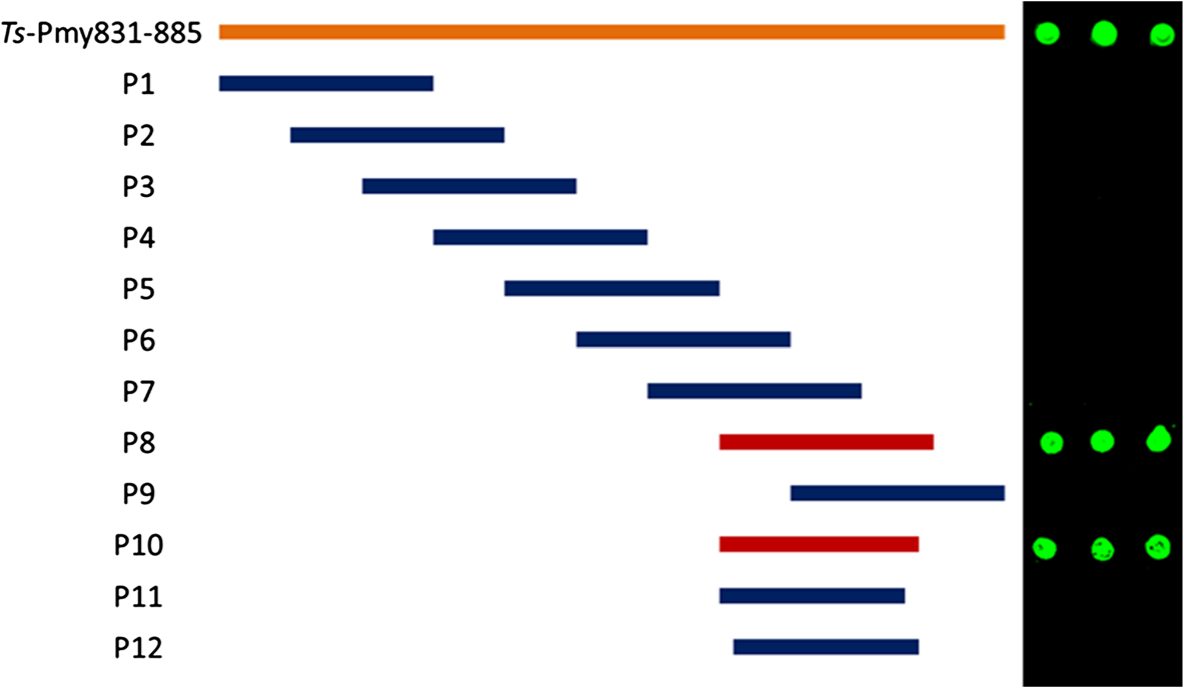
**Further mapping of the C9 binding site in overlapped synthesized peptides.** Different overlapped peptides within the C9 binding fragment of *Ts*-Pmy (Pmy831-885) were synthesized, and then spotted onto a nitrocellulose membrane by using a narrow-mouth pipette tip (5 μg in 2.5 μl). After being blocked with 1% BSA in PBS, the membrane was incubated with human C9 (1 μg/ml) at 37°C for 2 h and then with anti-C9 mAb (0.2 μg/ml) at room temperature for 1 h. IRDye 800CW-labeled goat anti-mouse IgG (50 ng/ml) was used as the secondary antibody. The peptide size diagram is shown on the left and the Dot-blot with C9 is shown on the right.

To further pinpoint the C9 binding site within the C-terminus of *Ts*-Pmy (Pmy831-885) defined by the fragment expressions, the 12 overlapped peptides covering this region were synthesized for their binding capacity to human C9 (Figure 3). After being spotted onto a nitrocellulose membrane, the synthesized peptide was probed with human C9 and detected with anti-C9 mAb. The Dot-blot analysis demonstrated that P8 within *Ts*-Pmy866-880 was able to bind to human C9 (Figure 3). The exact C9 binding sequence was narrowed down to *Ts*-Pmy866-879 (P10) by removing one amino acid at position 880. The further removal of one amino acid at the N-terminus (P11) or at the C-terminus (P12) caused the loss of the binding to human C9 (Figure 3), indicating that the sequence of the C9 binding site in *Ts*-Pmy was precisely narrowed down to 14 amino acid residues between ^866^Val to ^879^Met that forms the binding site structure to C9. As a non-relevant control, BSA did not bind to human C9.

### Inhibition of C9 polymerization by the binding site peptide P10

**Figure 4:**
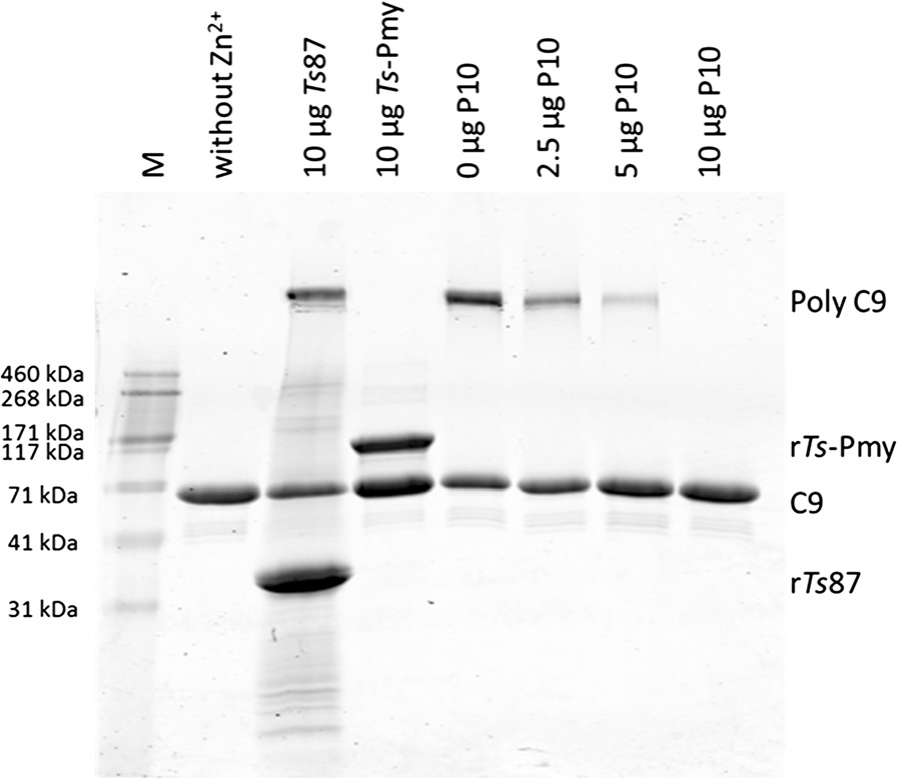
**Inhibition of Zn**^**2+**^**-induced C9 polymerization by the synthesized peptide P10.** To determine the effect of the synthesized peptide P10 on C9 polymerization, human C9 (3 μg) was pre-incubated with different amounts of peptide P10 (0 μg, 2.5 μg, 5 μg and 10 μg) at 37°C for 40 min and then incubated with 50 mM ZnCl_2_ in 20 mM Tris buffer (pH 7.2) at 37°C for 2 h. Blank C9 without adding Zn^2+^ was used as a negative control. The full-length r*Ts*-Pmy (10 μg) was used as positive control and *Ts*87 (10 μg) was used as non-relevant control. M, standard protein marker. The reaction mixtures were analyzed by SDS-PAGE.

To determine whether binding peptide P10 (^866^Val to ^879^Met) inhibits the Zn^2+^-induced C9 polymerization as full-length r*Ts*-Pmy [16], C9 was mixed with different amount of peptide P10 (2.5, 5, 10 μg) and then incubated with 50 mM ZnCl_2_. As shown in Figure 4, peptide P10 inhibited Zn^2+^-induced C9 polymerization in a dose-dependent manner. The polymerization of 3 μg human C9 was completely inhibited by 10 μg peptide P10 (Figure 4) and similarly by the full-length r*Ts*-Pmy. r*Ts*87 (10 μg) did not inhibit C9 polymerization as a non-relevant control. Without Zn^2+^, the C9 did not form the polymer (Figure 4).

### Inhibition of complement-mediated hemolysis by the binding site peptide P10

**Figure 5:**
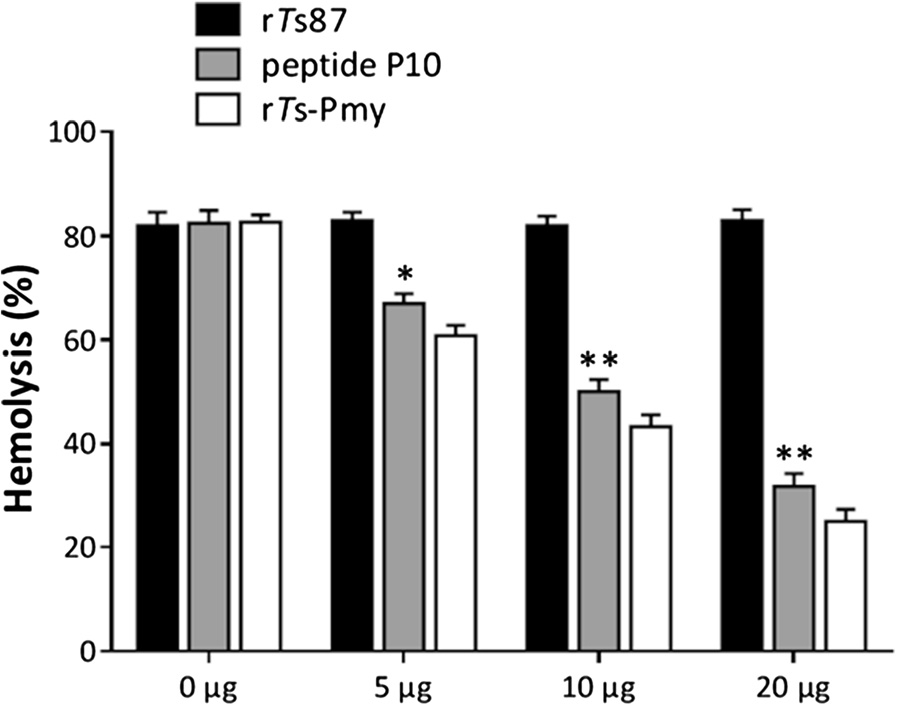
**Inhibition of complement-mediated hemolysis by peptide P10.** NHS was pre-incubated with various amounts (0, 5, 10, 20 μg) of peptide P10 and r*Ts*-Pmy in Mg-EGTA solution (5 mM MgCl_2_, 10 mM EGTA) for 30 min before adding the mixture into freshly washed E_R_ (1 × 10^8^) in 100 μl HBSS at 37°C for 30 min. r*Ts*87 was used as a non-relevant control. The amount of hemoglobin released into the supernatant was measured at 412 nm, and the percent lysis was calculated compared to the complete lysis (water). The results are shown as the means ± SE for three independent experiments. *p < 0.05. **p < 0.01.

After incubation with different amounts of peptide P10 and r*Ts*-Pmy (0, 5, 10, 20 μg), complement-mediated E_R_ lysis with NHS via the alternative pathway was significantly inhibited in a dose-dependent manner (Figure 5), which suggested that peptide P10 bound to C9 and consequently inhibited the complement-mediated hemolysis. As a non-relevant control, r*Ts*87 did not inhibit complement-mediated hemolysis.

## Discussion

The complement system has a critical role in the recognition, opsonization and elimination of pathogenic intruders. Among bacteria, viruses, fungi and parasites, many species have developed specific complement-evasion strategies to escape the attack from host’s immune system. The most common complement-evasion mechanism is to capture the soluble host complement regulators or express their structural mimics on the microbial surface [10]. Similar to other pathogens, helminths have a particularly wide and diverse arsenal of complement-evasion proteins, many of which have been characterized recently [10].

Paramyosin presents in thick myofilaments of invertebrate muscle and forms medullary rods surrounded by a cortical array of myosin rods [23]. In helminths, paramyosin serves not only as a structural protein but also as an immunomodulator. Earlier studies identified the expression of paramyosin in the tegument and on the surface of *Taenia solium*[24], *Schistosoma mansoni*, *Schistosoma japonicum*[25] and *Fasciola hepatica*[26]. It was observed that the paramyosin of helminth parasites could bind to human collagen [27-29], calgranulin [30], IgG [25,28,29], IgA [31], C1q [32], C8 [33] and C9 [29,33], suggesting that surface-exposed paramyosin may play an important role as a potential modulator of the host immune system.

Our previous results showed that *Ts*-Pmy is present on the outer membrane of the cuticle of the adults and NBL of *T. spiralis*[16]. It was confirmed that surface-exposed *Ts*-Pmy bound to complement C8 and C9, which are important components of the complement activation cascade and the membrane attack complex (MAC). The polymerization of C9, which was induced by Zn^2+^, was highly inhibited by r*Ts*-Pmy, indicating its interference in the assembly of the MAC during complement activation. The alternative complement pathway that activates the complement complex or polyC9 on rabbit erythrocytes (E_R_) was also inhibited by r*Ts*-Pmy. Native *Ts*-Pmy on the surface of *T. spiralis* effectively protected NBL from attack by the host complement system [14]. These results suggest that the outer membrane form of paramyosin expressed by *T. spiralis* had a role in host immunomodulation, presumably by inhibiting the formation of the MAC and thereby protecting the parasite from being attacked by the activated complement.

In this study, we further confirmed that native *Ts*-Pmy in the adult worm extracts was able to bind to human C9. In order to determine the C9 binding site on *Ts*-Pmy, different overlapped r*Ts*-Pmy fragments were expressed as recombinant proteins and their ability to bind to C9 was tested. The results showed that the C9 binding region was located at the C-terminus of *Ts*-Pmy within position of 571–885, further narrowed down to the region between ^831^Leu and ^885^Tyr. To further map the complement C9 binding site within *Ts*-Pmy831-885, we prepared ten synthesized peptides covering the sequence of *Ts*-Pmy831-885 with a few amino acids overlapping each other (P1-P12). The result of the binding assay demonstrated that 15-amino acid peptides P8 (866–880) strongly bound to human C9. Chopping off one amino acid at C-terminus (P10) kept the same binding ability to C9, indicating that the C9 binding site on *Ts*-Pmy consists of 14 amino acid residues (^866^Val-^879^Met). Chopping off either ^866^Val (P12) or ^879^Met (P11) lost the binding ability to C9, suggesting that both residues at both ends of the binding site, ^866^Val and ^879^Met, are necessary for the binding affinity to human C9. A similar mapping study for the C9 binding site on paramyosin of *Schistosoma mansoni* (*Sm*-Pmy) only narrowed down to 123 amino acid residues in the C-terminus [34]. Up to date, it is the first report to precisely locate the C9 binding site of paramyosin in helminths.

Functional analysis in this study revealed that peptide P10 (^866^Val-^879^Met) was comparable to bind to C9 and further inhibit its function as full-length r*Ts*-Pmy including C9 polymerization and complement-mediated E_R_ lysis, suggesting that the binding site peptide could effectively inhibit the assembly of MAC and therefore protect the parasite from being attacked. Such results were consistent with the observations in the study on *Sm*-Pmy, which showed that C9 polymerization and hemolytic activity of human complement was inhibited by the C-terminal region of *Sm*-Pmy [34]. Therefore, the C-terminal regions of *Ts*-Pmy and *Sm*-Pmy have similar functions as complement inhibitors in capturing the complement component C9 to evade the first line of host’s immune defense as a survival strategy. *T. spiralis* produces *Ts*-Pmy through a 14 amino acids binding site to bind and neutralize C9 as a direct complement inhibitor.

r*Ts*-Pmy has been evaluated as a potential vaccine candidate antigen due to its complement neutralizing function [13,14]. The finding of the C9 binding domain on *Ts*-Pmy provides a good therapeutic target and a feasible approach to develop an epitope-based subunit vaccine against trichinellosis. A monoclonal antibody against the C9 binding domain has been produced and primary data with the antibody demonstrated protective immunity in passively transferred mice against *T. spiralis* larval infection (data not shown), indicating its potential as an epitope vaccine.

Our previous study revealed that another two epitopes on *Ts*-Pmy recognized by protective monoclonal antibodies, one of which is located between ^88^Glu and ^107^Glu at the N-terminus of *Ts*-Pmy and another is a conformational epitope without a specific location, were also protective in immunized mice against *T. spiralis* larval challenge [19], indicating *Ts*-Pmy may play multiple functions in the parasite life cycle, except for acting as an immunomodulator through neutralizing complement. A multi-epitope vaccine based on these two *Ts*-Pmy epitopes and another protective epitope from *Ts*87 produced higher levels of protection compared to the individual epitope vaccine [16]. However, the protection induced by these epitopes is not complete (~35%). The addition of the C9 binding epitope identified in this study into the multiple epitope pipelines may increase the efficacy of protection against *T. spiralis* infection. The protective immunity of the *Ts*-Pmy C9 binding domain and its combination with other identified epitopes from *T. spiralis* vaccine pipelines against *T. spiralis* infection is under investigation.

## Conclusions

This study mapped the C9 binding domain at the C-terminus between ^866^Val and ^879^Met of vaccine antigen *Ts*-Pmy, which was capable of binding to C9 and preventing C9 polymerization. Our results revealed the precise molecular basis for *T. spiralis* utilizing *Ts*-Pmy as an immunomodulator to resist the attack of the host complement system. These results provide molecular evidence that *T. spiralis* evades host complement attacks by capturing and neutralizing complement components. This functional understanding of complement-evasion could serve as an important starting point for the development of site-targeting therapeutics and epitope-based subunit vaccines.

## Competing interests

The authors declare that they have no competing interests.

## Authors’ contributions

XZ performed the experiments and drafted the manuscript. YWH, JY, and YG performed some of the experiments. XPZ designed the study and revised the manuscript. All authors read and approved the final manuscript.
